# Simplified Zenker’s diverticulum endoscopic myotomy performed with a new bipolar scissor device

**DOI:** 10.1055/a-2615-1338

**Published:** 2025-07-01

**Authors:** Eduardo Albéniz, Zacharias Tsiamoulos, Roser Vega, Shi Jie Looi, Fermín Estremera-Arevalo, Marta Gómez Alonso, Sauid Ishaq

**Affiliations:** 1 Gastroenterology Department, Department, Hospital Universitario de Navarra (HUN); Navarrabiomed; Universidad Pública de Navarra (UPNA); IdiSNA, Navarra, Spain; 2 2241 Gastroenterology Department, East Kent Hospitals University NHS Foundation Trust, Canterbury, United Kingdom; 3 8964 Gastroenterology Department, University College London Hospitals, NHS Trust, London, United Kingdom; 4 7714 Dudley Group of Hospitals NHS Trust, Birmingham, United Kingdom

Zenker’s diverticulum (ZD) is a pulsion diverticulum due to abnormal relaxation of the cricopharyngeal muscle (CPM). Several modifications of the flexible endoscopic myotomy technique (Z-POEM) have been reported 1 2 .

We present the case of a 51-year-old man with symptomatic Zenker’s diverticulum ( Video 1 ). This was treated by innovative adaption of the flexible endoscopic myotomy technique (Z-POEM) by using a novel scissor-knife device called *Spyderblade* (SB), which operates on an electrosurgical system utilizing bipolar radiofrequency energy for cutting and super-high-frequency microwaves for coagulation (Croma platform). We performed a small mucosotomy ( Fig. 1 ) over the septum (smaller than the plastic cap size). The distal SB jaw acts as a tip knife for the initial dissection, transitioning to a scissor mode for other steps.

Simplified Z-POEM performed using a novel bipolar scissor-knife device utilizing bipolar radiofrequency energy for cutting and super-high-frequency microwaves for coagulation.Video 1

**Fig. 1:**
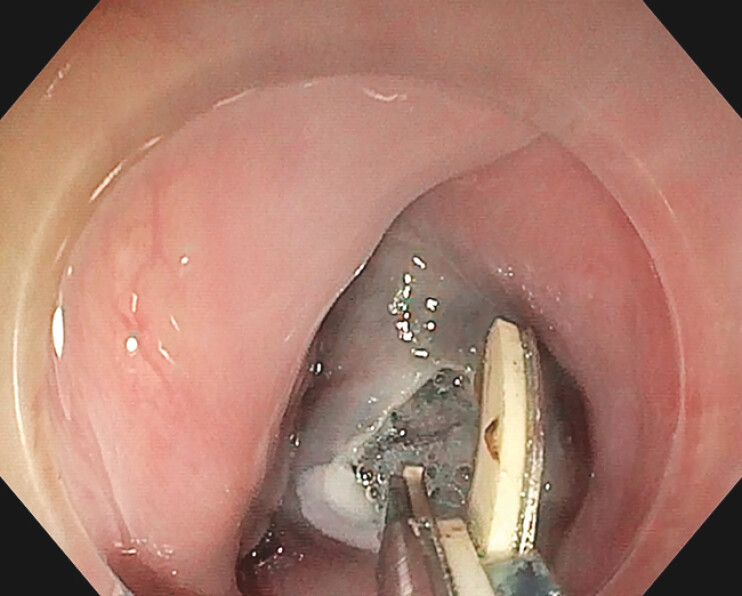
Initial mucosotomy.

We began the myotomy on the exposed muscle, relaxing the tension in the area and enabling entry into the submucosal space. Without performing any lateral tunneling, a complete myotomy of the cricopharyngeal muscle (CPM) and the initial esophageal muscularis propria was achieved ( Fig. 2 ). Small amount of injection was needed.

**Fig. 2:**
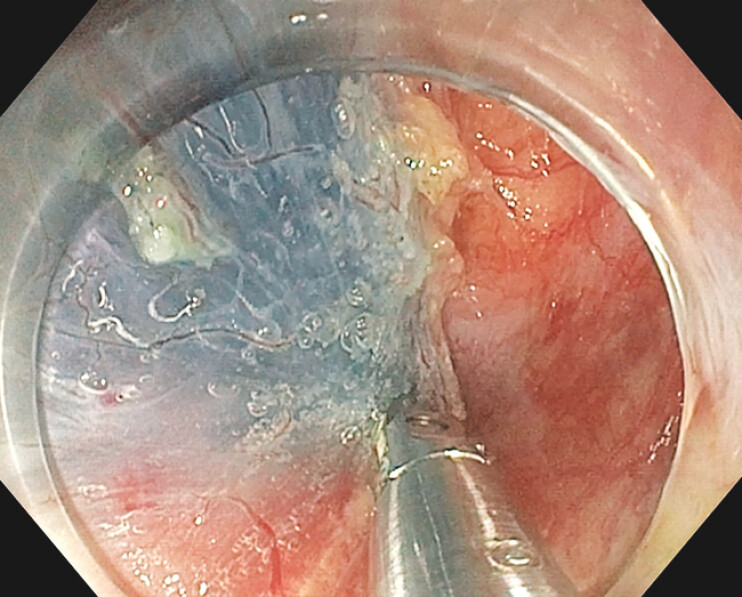
Complete myotomy.

The scissor-type knife isolation of both external jaw sides minimizes the risk of surrounding structures damage. Small vessels were coagulated using the active jaw tip, while larger vessels were addressed in scissor mode offering precise control.

Following the myotomy, a central longitudinal cut extended to the diverticular fundus to complete mucosotomy ensuring the residual mucosal pouch is fully addressed ( Fig. 3 ). Additional lateral mucosotomies were performed as required based on the size of the ZD. Finally, short-stem clips were used to close the entire defect ( Fig. 4 ).

**Fig. 3:**
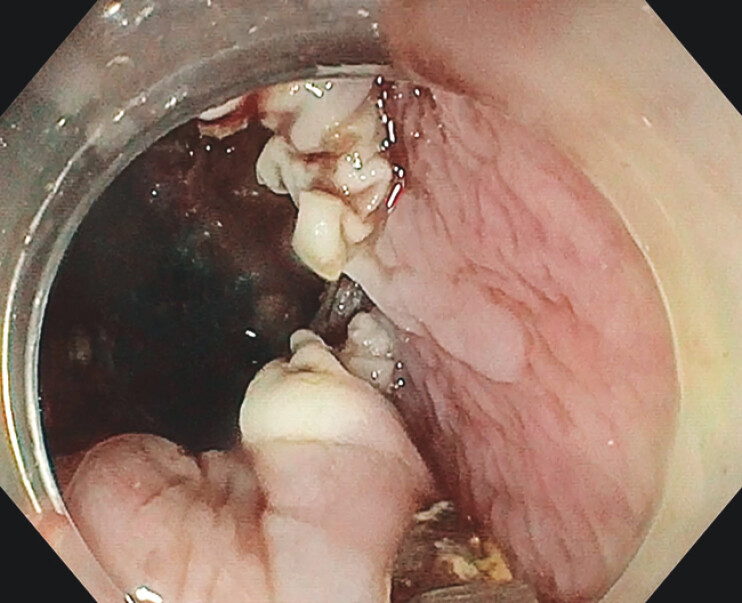
Complete pouch mucosotomy.

**Fig. 4:**
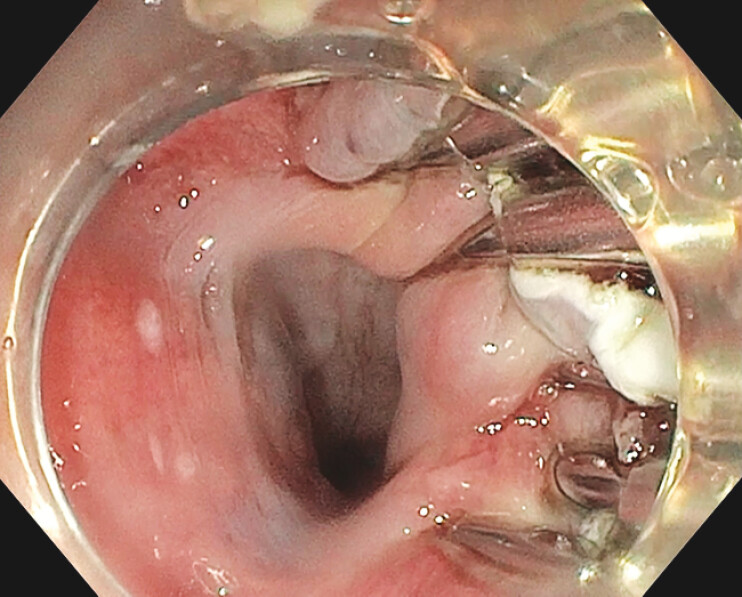
Clip closure from esophageal lumen.

In conclusion, minor technical modifications simplify the Z-POEM technique, and the incorporation of bipolar technology reduces the risk of complications and can be used in patients with implantable devices. This approach could make the Z-POEM technique more accessible to endoscopists with less experience in third-space endoscopic procedures.

Endoscopy_UCTN_Code_TTT_1AO_2AP

## Notes

Endoscopy E-Videos https://eref.thieme.de/e-videos
*E-Videos* is an open access online section of the journal *Endoscopy* , reporting on interesting cases and new techniques in gastroenterological endoscopy. All papers include a high-quality video and are published with a Creative Commons CC-BY license. Endoscopy E-Videos qualify for HINARI discounts and waivers and eligibility is automatically checked during the submission process. We grant 100% waivers to articles whose corresponding authors are based in Group A countries and 50% waivers to those who are based in Group B countries as classified by Research4Life (see: https://www.research4life.org/access/eligibility/ ). This section has its own submission website at https://mc.manuscriptcentral.com/e-videos .
